# The expanding clinical spectrum of autoinflammatory diseases with *NOD2* variants: a case series and literature review

**DOI:** 10.3389/fimmu.2024.1342668

**Published:** 2024-01-29

**Authors:** Anastasios Karamanakos, Olga Vougiouka, Evdoxia Sapountzi, Aliki I. Venetsanopoulou, Maria G. Tektonidou, Anastasios E. Germenis, Petros P. Sfikakis, Katerina Laskari

**Affiliations:** ^1^ Joint Academic Rheumatology Program, First Department of Propaedeutic and Internal Medicine, National and Kapodistrian University of Athens, Athens, Greece; ^2^ Department of Rheumatology, Evangelismos General Hospital, Athens, Greece; ^3^ Second Department of Pediatrics, National and Kapodistrian University School of Medicine, “P. A. Kyriakou” Children’s Hospital, Athens, Greece; ^4^ Second Department of Pediatrics, School of Medicine, Faculty of Health Sciences, Aristotle University of Thessaloniki, American Hellenic Educational Progressive Association (AHEPA) University General Hospital, Thessaloniki, Greece; ^5^ Rheumatology Clinic, Department of Internal Medicine, Medical School, University of Ioannina, Ioannina, Greece; ^6^ Department of Immunology and Histocompatibility, School of Medicine, University of Thessaly, Larissa, Greece

**Keywords:** *NOD2*, systemic autoinflammatory disease, Yao syndrome, coexisting gene variants, NGS

## Abstract

**Objective:**

To assess the impact conferred by *NOD2* variants on the clinical spectrum of patients with systemic autoinflammatory diseases (SAIDs) in Greece.

**Methods:**

Consecutive patients (n=167) with confirmed SAIDs who underwent screening by next generation sequencing (NGS) targeting 26 SAID-associated genes, and carried at least one *NOD2* gene variant, were retrospectively studied. The demographic, clinical and laboratory parameters were recorded.

**Results:**

In total, 24 rare *NOD2* variants in 23/167 patients (14%) were detected. Notably, 18 patients had at least one co-existing variant in 13 genes other than *NOD2*. Nine patients had juvenile- and 14 adult-onset disease. All patients presented with symptoms potentially induced by the *NOD2* variants. In particular, the candidate clinical diagnosis was Yao syndrome (YAOS) in 12 patients (7% of the whole SAID cohort). The clinical spectrum of patients with YAOS (mean episode duration 8 days) was fever (n=12/12), articular symptoms (n=8), gastrointestinal symptoms (n=7; abdominal pain/bloating in 7; diarrhea in 4; oral ulcers in 3), serositis (n=7), and rash (n=5), while the inflammatory markers were elevated in all but one patient. Most of these patients showed a poor response to nonsteroidal anti-inflammatory drugs (n=7/9), colchicine (n=6/8) and/or anti-TNF treatment (n=3/4), while a complete response was observed in 6/10 patients receiving steroids and 3/5 on anti-IL1 treatment. Another 8 patients were diagnosed with either FMF (n=6) or PFAPA syndrome (n=2) presenting with prominent diarrhea (n=7), oral ulcers (n=2), periorbital swelling and sicca-like symptoms (n=1), or maculopapular rash (n=1). One patient had a clinically undefined SAID, albeit characterized by oral ulcers and diarrhea. Finally, one patient presented with chronic relapsing urticaria with periorbital edema and inflammatory markers, and another one had a Crohn-like syndrome with good response to anti-IL-1 but refractory to anti-TNF treatment.

**Conclusion:**

*NOD2* variants were detected in 1 out of 7 SAID patients and seem to have an impact on disease phenotype and treatment response. Further studies should validate combined molecular and clinical data to better understand these distinct nosological entities.

## 1 Introduction

Systemic autoinflammatory diseases (SAIDs) are a group of genetically heterogenous disorders presenting as sterile, episodic and unprovoked inflammatory attacks driven by the innate immune system. Characteristically, SAIDs lack the high autoantibody titers and autoreactive T cells characterizing autoimmune disorders (1, 2). Historically they are classified as monogenic autoinflammatory disorders (mAIDs), inherited in a mendelian pattern, and polygenic, in which no straightforward pattern of inheritance is observed (3). Interestingly, a new concept in genomic medicine termed genetically transitional disease (GTD) seems to better define Yao syndrome (YAOS) (4), a *NOD2*-associated AID (NAID), linked to the nucleotide-binding oligomerization domain containing 2 (*NOD2*) gene, encoding a cytosolic NOD-like receptor (NLR) and innate immune sensor (Online Mendelian Inheritance in Man [OMIM] 617321) (5). According to this novel nomenclature, GTDs are conditions where mutations are necessary, but not sufficient alone to cause disease (4). YAOS represents a new disease entity with still expanding phenotypic and genotypic spectrum (6). It is linked to specific *NOD2* sequence variants, which are distinct from those described in Blau syndrome and early onset sarcoidosis, representing the familial and sporadic forms of the same pediatric noncaseating granulomatous SAID, respectively (7, 8), but also in multifactorial Crohn’s disease (9); on the other hand, there is also evidence supporting the presence of shared variants among these nosological entities. At the same time, *NOD2* variants have been shown to influence the phenotype of well-defined mAIDs such as Familial Mediterranean Fever (FMF) (10) or other SAIDs (11–28). Due to insufficient awareness, overlapping features and/or lack of extensive genetic screening, NAIDs are often underrecognized or described as a syndrome of undifferentiated recurrent fever (29). Nevertheless, in the era of next generation sequencing (NGS), the ability to screen multiple genes simultaneously has provided significant impact on diagnosing SAIDs and guiding therapy (30). Indeed, recent studies highlight the high frequency of *NOD2* variants and diagnosis of YAOS among patients with SAIDs (31). In this respect, we retrospectively studied patients with SAIDs who underwent genetic screening by NGS and carried at least one *NOD2* gene variant, in order to describe their genotypic and phenotypic characteristics, and provide evidence on the role of *NOD2* variants on SAID phenotypes in a Greek patient cohort.

## 2 Patients and methods

### 2.1 Study population

Consecutive patients (n=167) with SAIDs who underwent genetic screening by NGS targeting 26 SAID-associated genes (2015–2023) and carried at least one *NOD2* gene variant (n=23), were retrospectively studied. Medical information on demographic characteristics, clinical symptoms, laboratory parameters, and treatment details was retrieved from medical charts and a personal interview. A standard questionnaire was completed for each patient. The same physician in the Rheumatology Unit of the First Department of Propaedeutic Internal Medicine at the University of Athens (center of excellence for rare rheumatic diseases) confirmed disease diagnosis in all patients. The following classification/diagnostic criteria for autoinflammatory syndromes were recorded: Eurofever/Printo classification criteria for mAIDs and PFAPA (1), Yamaguchi diagnostic criteria for Still’s disease (32), YAOS diagnostic criteria (33). Good response to treatment was defined as complete resolution of clinical and biological disease-related manifestations, whereas partial response as any improvement without full recovery. The study was approved by the Ethics Committee of the National and Kapodistrian University of Athens. Informed consent was obtained from each patient or her/his legal guardians for participation in the study according to the Declaration of Helsinki. All data will be made available on request.

### 2.2 Genetic screening

Genetic screening was performed as previously described (34) at the Department of Immunology & Histocompatibility, University of Thessaly Medical School, in order to identify variants in the coding regions of 26 genes associated with SAIDs (**Supplementary Table 1**).

### 2.3 Variant classification

Variants with a worldwide frequency of >1% (1000 Genomes Global Minor Allele Frequency, ExAC) and polymorphisms (UCSC Common SNPs) for which no disease associations are reported in the public ClinVar disease database (https://www.ncbi.nlm.nih.gov/clinvar/), as well as synonymous single-nucleotide variants (SNVs) were excluded from further analysis. To assess the pathogenicity of variants, the ClinVar and INFEVERS databases (https://infevers.umai-montpellier.fr/web/) as well as Varsome, an aggregator and impact analysis tool for human genetic variation (https://varsome.com/about/general/varsome-citations/), were used in addition to literature search. We classified genetic variants as likely benign, benign, VUS (variant of uncertain significance), likely pathogenic or pathogenic for a certain disease, according to the ACMG guidelines and Eurofever criteria (1, 35).

### 2.4 Disease classification

After considering both clinical findings and genetic results, each patient got a final diagnosis. The genetic results supported a final diagnosis if the patient had clinical symptoms compatible with this diagnosis. A definite disease diagnosis was set if clinical signs and symptoms were compatible with a single SAID, especially in the presence of a confirmatory or a non-confirmatory genotype (35). The disease diagnosis was considered undefined when a particular diagnosis could not be made. If the clinical and genetic characteristics were compatible with more than one SAIDs, the diagnosis of an overlap or mixed syndrome has been suggested. According to the diagnostic criteria for YAOS (33), identification of certain *NOD2* variants, including rare ones, was required for this diagnosis.

## 3 Results

**Figure 1:**
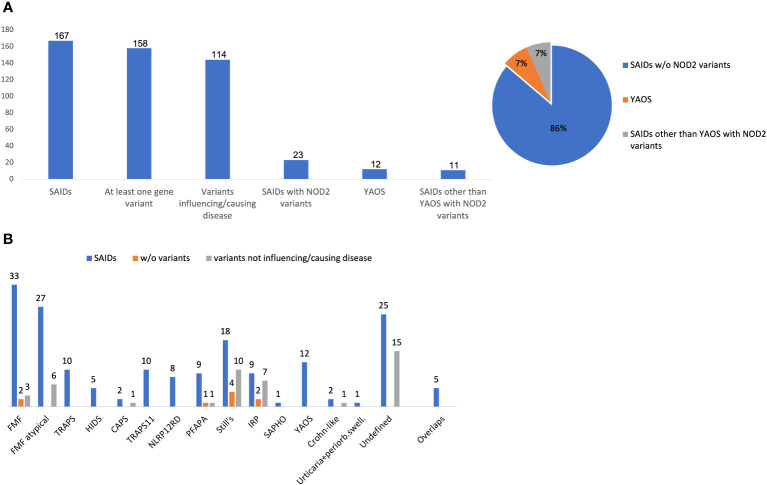
**(A)** Frequency of *NOD2* variants, Yao syndrome or other diagnoses among 167 patients with systemic autoinflammatory diseases. **(B)** Disease diagnosis (blue bar), patients without variants (orange bar) or carrying only variants not influencing/causing disease (grey bar) in each disease. The y-axis indicates the absolute number of patients. Variants not influencing/causing disease are defined as non-contributory genotypes in *MEFV*, *TNFRSF1A*, *MVK*, *NLRP3*, *NLRP12* (Eurofever criteria) (1), and/or variants in other genes not related to phenotype.Overlap diagnoses in 5 patients include the following: FMF/TRAPS11, n= 3; TRAPS/TRAPS11, n=1; HIDS/NLRP12RD, n=1. w/o, without; SAID, systemic autoinflammatory disease; YAOS, Yao syndrome; FMF, Familial Mediterranean Fever; PFAPA, Periodic Fever, Aphthous Stomatitis, Pharyngitis, Adenitis; *NLRP12* RD, *NLRP12* related disease; CAPS, cryopyrin-associated periodic syndrome; HIDS, Hyperimmunoglobulinemia D with periodic fever syndrome; TRAPS, Tumor necrosis factor receptor-associated periodic syndrome; TRAPS11, *TNFRSF11A* associated hereditary fever disease; IRP, idiopathic relapsing pericarditis; SAPHO, Synovitis, Acne, Pustulosis, Hyperostosis, and Osteitis; periorb. swell, periorbital swelling.

**Figure 2:**
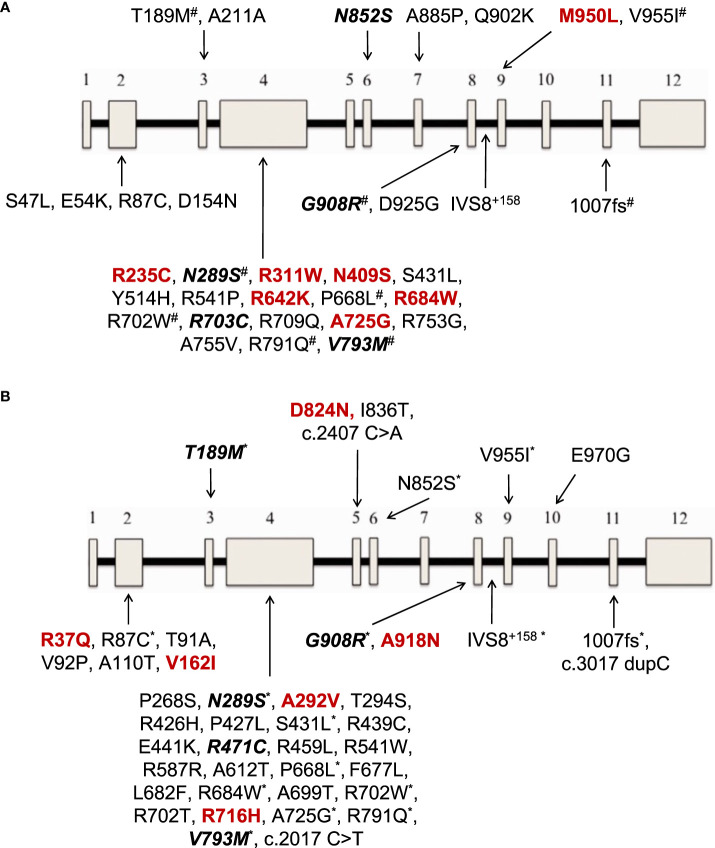
**(A)** *NOD2* gene variants reported in 12 patients with Yao syndrome in the present study and/or in literature. **(B)** *NOD2* gene variants reported in patients with diagnoses other than Yao syndrome in 11 patients in the present study and/or in literature. In colored font are depicted variations found only in our series, in bold italics font those found both in our cohort and in literature, and the rest (regular font) are variants reported only in literature. The 12 exons of the gene are depicted as boxes, and the black line connecting the exons represents the intronic gene regions. ^#^variants also found in patients with diagnoses other than Yao syndrome ^*^variants also found in patients with Yao syndrome.

**Figure 3:**
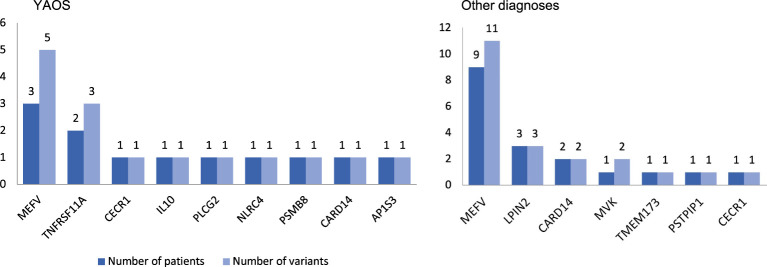
Coexisting variants in genes other than *NOD2* in 8 out of 12 patients with Yao syndrome (YAOS) (67%) and 10 out of 11 patients with other diagnoses (91%). The y-axis indicates the absolute number of patients (dark blue bars)/variants (light blue bars).

At least one *NOD2* variant was detected in 14% of SAID patients (23 out of 167) (**Figure 1A**). In particular, 15% of 158 patients with positive genetic testing and 20% of 114 patients with variants possibly influencing/causing disease, carried *NOD2* variants. Among these 23 patients, 17 were adults (74%) and 6 children; 14 patients (61%) had adult-onset and 9 juvenile-onset disease. Thirteen of them (57%) were females. All were of Greek origin except for three patients of Turkish, Armenian or Arabic descent. Overall, variants were found in 14 out of 26 SAID-associated genes screened (*NOD2* included). In total, 24 rare *NOD2* (**Figures 2A, B**) and 36 variants in the other genes were detected; 18 out of 23 patients had at least one variant located in 13 genes other than *NOD2*. Among these 13 genes the most commonly affected was *MEFV* (16 variants in 12 patients), followed by *TNFRSF11A* (3 variants in 2 patients) and *LPIN2* (3 variants in 3 patients) (**Figure 3**). A detailed report of patients’ genotype is provided in **Supplementary Table 2**.

### 3.1 Characteristics of patients fulfilling criteria for YAOS

Twelve out of 23 patients carrying *NOD2* variants (52%) received the diagnosis of YAOS (7% of the whole cohort). YAOS was the most frequent diagnosis among patients with *NOD2* variants and the fourth in order of frequency SAID in our whole cohort following FMF, unclassified disease and Still’s disease (**Figures 1A, B**). The detailed patient characteristics are shown in **Table 1**; **Supplementary Table 2**. The slight majority of patients (58%) was male, and all of them were adults at disease onset (mean age 36 years), with a mean disease duration of 6.3 ± 6.5 years at the time of assessment. The most common symptom was high grade fever, which was present in all patients and had an intermediate duration of 6.5 ± 4.7 days. Most of the patients had almost monthly episodes (the mean number of attacks per year was 13). Polyarthritis/arthralgia was the second common symptom (67%), while approximately 2/3 had gastrointestinal involvement, mainly with abdominal pain, and 1/4 had oral ulcers. One patient was found to have terminal ileitis in colonoscopy but without Crohn’s disease findings in biopsy. Serositis was present in 58% of patients, myalgias in 33%, and almost 1/4 had a maculopapular or urticarial rash. One patient suffered from sicca symptoms, and one presented with lower leg swelling. All but one patient had increased inflammatory markers during episodes. With regard to genetic analysis, all patients were single heterozygous except for one compound heterozygous *NOD2* genotype (**Supplementary Table 2**). Eight patients had 15 variants in genes other than *NOD2* (**Figure 3**); most frequently *MEFV* (in total 3 pathogenic and 2 VUS variants in 3 patients) and *TNFRSF11A* (3 variants in 2 patients), but also *NLRC4*, *CECR1*, *IL10*, *PLCG2*, *AP1S3*, *PSMB8* and *CARD14*.

**Table 1:** Characteristics of 12 patients with Yao syndrome in the present study.

Characteristics		mean ± SD *or* N (%)
Demographics	Males/Females	7 (58)/5 (42)
	Age, *years*	36 ± 10.2
	Juvenile/adult-onset disease	-/12 (100)
	Disease Duration, *years*	6.3 ± 6.5
Clinical findings	Attack Duration, *days*	8.3 ± 5.7
	Attack frequency/*year*	13 ± 15.2
	Fever/>38°C	12 (100)/9 (75)
	Fever duration, *days*	6.5 ± 4.7
	Articular symptoms	8 (67)
	Myalgias	4 (33)
	Periorbital oedema	3 (25)
	Rash	5 (42)
	- maculopapular	2 (17)
	- urticaria	1 (8)
	- psoriasiform	1 (8)
	- pustulosis	1 (8)
	Oral ulcers	3 (25)
	Serositis	7 (58)
	- Pleuritis	4 (33)
	- Pericarditis	6 (50)
	Gastrointestinal involvement	7 (58)
	- Abdominal pain/diarrhea	7 (58)/4 (33)
	- Crohn-like findings (colonoscopy)	1 (8)
	Sicca symptoms	1 (8)
	Lower limb edema	1 (8)
Laboratory parameters	Leukocytosis/Î PMNs	7 (58)/8 (67)
	Anemia	3 (25)
	Thrombocytosis	3 (25)
	Elevated inflammatory markers	11 (92)
	Elevated SAA	4/5 (80)
Treatment (good response)	NSAIDs	2/9 (22)
	GC	6/10 (60)
	Colchicine	2/8 (25)
	Sulfasalazine	0/2
	Anti-IL-1	3/5 (60)
	Anti-TNFa	1/4 (25)

PMNs: polymorphonuclear cells; SAA: serum amyloid A; NSAIDs: non-steroidal anti-inflammatory drugs; GC: glucocorticoids; SSZ: sulfasalazine; IL: interleukin; TNF: tumor necrosis factor.

The co-existing variants in genes other than *NOD2* might have an impact on disease phenotype in 4 patients with YAOS with features of mAIDs [*TNFRSF11A*-associated hereditary fever disease - TRAPS11 (36–38), n=2 and/or FMF, n=3] (**Supplementary Table 2**). In particular, patient no. 10 in **Supplementary Table 2**, had a pathogenic variant in the *MEFV* gene and a rare variant in *TNFRSF11A*. The patient presented with recurrent periodic episodes of high fever lasting 72 hours, accompanied by serositis, periorbital edema, and maculopapular rash, with good response to steroids. Another patient (no. 11 in **Supplementary Table 2**) had two rare variants in the *TNFRSF11A* gene as well as a variant in *PSMB8*. Clinical features included fever episodes lasting up to 10 days, pericarditis, abdominal pain, splenomegaly, partial response to non-steroidal anti-inflammatory drugs (NSAIDs) and colchicine, albeit good response to steroids. Finally, two other patients with pathogenic *MEFV* variants (patients no. 1 and 2 in **Supplementary Table 2**), the first showed a good response to colchicine, while the second one had one-day fever episodes.

Overall, patients with YAOS showed a poor response to NSAIDs (n=7/9), colchicine (n=6/8) and/or anti-TNFa treatment (n=3/4), while more than half of the patients receiving steroids (n=6/10) and most of the patients receiving anti–IL-1 agents (n=3/5) had a complete resolution of symptoms and normalization of laboratory parameters. Lastly, two patients received sulfasalazine and one of them showed a partial improvement of his gastrointestinal symptoms.

### 3.2 Characteristics of patients with diagnoses other than YAOS

**Figure 4:**
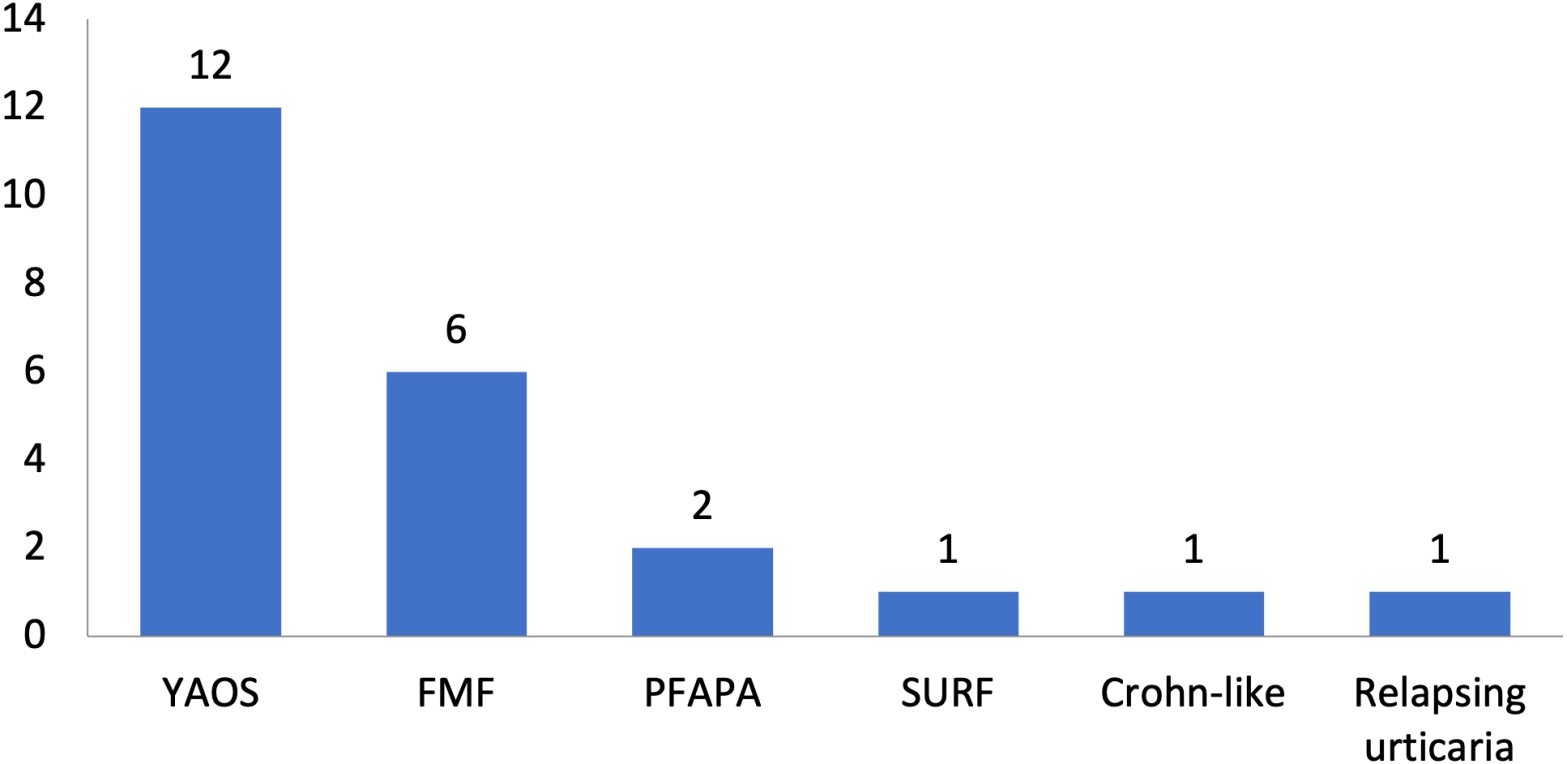
Disease diagnoses among 23 patients with systemic autoinflammatory diseases and rare *NOD2* variants. Twelve patients had Yao syndrome (52%) and 11 other diagnoses (48%). YAOS, Yao syndrome; FMF, Familial Mediterranean Fever; PFAPA, Periodic Fever, Aphthous Stomatitis, Pharyngitis, Adenitis; TRAPS11, *TNFRSF11A* associated hereditary fever disease; SURF, syndrome of undifferentiated recurrent fever.

In total, 11 patients with SAIDs and diagnoses other than YAOS carried *NOD2* variants (48% of all patients carrying *NOD2* variants and 7% of the whole cohort) (**Figures 1A**, **4**). In particular, we found a frequency of 7% among patients with positive genetic testing, while 10% among patients with variants possibly influencing/causing disease. The characteristics of non-YAOS patients are summarized in **Table 2** and shown in detail in **Supplementary Table 2**. Most of the patients with *NOD2* gene variants and a disease diagnosis other than YAOS were females (n=8), they had juvenile-onset disease (n=9) and a mean age of 8 years at first symptom. All patients were heterozygous for *NOD2* variants (**Figure 2B**) and all but one, had at least one variant in 7 other SAID-associated genes. Among patients with other gene variants, all but one had at least one variant in the *MEFV* gene (11 variants in 9 patients), whereas fewer patients in *LPIN2* (3 variants in 3 patients), *CARD14* (2 variants in 2 patients), *MVK* (2 variants in 1 patient), *TMEM173*, *PSTPIP1*, and *CECR1* (**Figure 3**).

**Table 2:** Characteristics of 11 patients with *NOD2* variants and diagnoses other than Yao syndrome in the present study.

Characteristics		mean ± SD *or* N				
Diagnosis		FMF n=6*	PFAPA n=2	SURF n=1	Crohn-like n=1	Relapsing urticaria n=1
Demographics	M/F	1/5	1/1	F	M	F
	Age at disease onset	4.3 ± 3.4	4.5 ± 3.5	4	31	23
	Juvenile/adult-onset	Juvenile	Juvenile	Juvenile	Adult	Adult
	Disease Duration, *yrs*	22 ± 22	3 ± 2	3	10	5
Clinical findings	Attack Duration, *days*	3 ± 2	3.5 ± .5	3	15	1
	Attack frequency/*yr*	7 ± 3.5	11 ± 1	12	3	48
	Fever/>38°C	6/6	2/2	Yes/Yes	No	No/No
	Fever duration, *days*	2.5 ± .5	3.5 ± .5	3	–	–
	Articular symptoms	3	1	No	Yes	Yes
	Myalgias	No	1	Yes	Yes	No
	Periorbital oedema	1	No	No	No	Yes
	Rash	4	No	Yes	No	Yes
	- maculopapular	2		–		–
	- erysipelas-like	1		–		–
	- urticaria	1		Yes		Yes
	Oral ulcers	1	1	Yes	No	No
	Serositis	3	No	Yes	No	No
	- Pleuritis	2		Yes		
	- Pericarditis	2		Yes		
	Gastrointestinal involvement	6	2	Yes	Yes	No
	- Abdominal pain/diarrhea	6/5	2/2	Yes/Yes	Yes/Yes	–
	- Crohn-like findings (colonoscopy)	NA	NA	NA	Yes	–
	Sore throat	2	2	No	No	No
	Nephritis	1	No	No	Yes^#^	No
	Lymphadenopathy	1	2	No	Yes	No
	Organomegaly	1	No	No	Yes	No
Laboratory parameters	Leukocytosis/Î PMNs	3/3	1/2	No/No	Yes/Yes	No/No
	Anemia	1	1	No	Yes	No
	Elevated inflammatory markers	3	2	Yes	Yes	Yes
	Elevated SAA	3	1	NA	Yes	No
Treatment response	NSAIDs	1	1	NA	–	–
	GC	–	1	NA	Yes	No
	Colchicine	3	1	NA	–	–
	Anti-IL-1	1	No	NA	Yes	–
	Anti-TNFa	1	No	NA	No	–

*laboratory findings available in 5 patients, treatment information available in 4 patients; # focal glomerulosclerosis.

Yr, year; FMF, Familial Mediterranean Fever; PFAPA, Periodic Fever, Aphthous Stomatitis, Pharyngitis, Adenitis; SURF, syndrome of undifferentiated recurrent fever; M, male; F, female; PMNs, polymorphonuclear cells; LFTs, liver function tests; SAA, serum amyloid A; NSAIDs, non-steroidal anti-inflammatory drugs; GC, glucocorticoids; IL, interleukin; TNF, tumor necrosis factor; NA, not available.

All patients presented with symptoms potentially induced by variants in the *NOD2* gene. In particular, among 6 patients receiving the diagnosis of FMF, 5 presented with prominent diarrhea, of which one had also oral ulcers and showed a poor response to colchicine, while one developed periorbital swelling and sicca-like symptoms later during disease course; the sixth patient, being compound heterozygous for two pathogenic *MEFV* variations, exhibited an extensive maculopapular rash (**Supplementary Table 2**). Another two patients fulfilled the criteria for PFAPA syndrome, albeit, they exhibited prominent diarrhea (n=2) and oral ulcers (n=1). One patient was diagnosed with an undifferentiated SAID (syndrome of undifferentiated recurrent fever – SURF); she presented with fever, myalgias, urticarial rash, prominent recurrent oral ulcers and diarrhea. Finally, one patient carrying the G908R *NOD2* variant, presented with a clinical syndrome resembling Crohn’s disease, albeit he was refractory to anti-TNFa treatment and showed good response to anti-IL-1 agents, and another one had relapsing urticaria refractory to steroids, periorbital swelling, and increased inflammatory markers.

## 4 Discussion

*NOD2* variants seem to be common when screening patients with SAIDs. Indeed, since the first mention of the *NOD2* gene on chromosome 16q12 and its protein in 2001 (39), up to 187 *NOD2* variants have been reported in Infevers database (https://infevers.umai-montpellier.fr/web/search.php?n=6). In our Greek cohort with SAIDs, 14% of patients carried *NOD2* variants; 15% of cases with positive genetic testing and 20% of those with variants possibly influencing/causing disease. More than half of these patients fulfilled the YAOS disease criteria (7% of the whole cohort). In agreement with our study, in a Chinese population with monogenic or polygenic SAIDs, Hua et al. detected *NOD2* variants in 10 out of 68 patients (15%); one third of them suffered from YAOS (4% of the whole cohort) (24), whereas Qin et al. reported 6 out of 80 (8%) patients with *NOD2* variants, all were finally diagnosed with YAOS (40). In an aforementioned study including 143 patients with clinical phenotypes suspicious for *NOD2*-associated disease, almost half of them (47%) carried *NOD2* variants. Among them, four-fifths received the diagnosis of YAOS (38% of the whole cohort) (41).

YAOS, first described in 2011 in 7 patients in the United States (3), seems to be more common than originally thought, especially when compared to other SAIDs (31), with an estimated prevalence of 1–10/100,000 (41). Indeed, YAOS was the fourth in order of frequency SAID in our whole cohort following FMF, unclassified disease and Still’s disease, and the most frequent diagnosis among patients with *NOD2* variants (52%), as also previously described (20). According to the largest studies (20, 33, 41, 42), YAOS is principally sporadic and is predominantly reported in white adults with a female-to-male ratio of 2:1; the disease occurs at any age, however, it is most common between age 20 and 50 (6), and recurrent episodes last from days to several weeks alternating with asymptomatic periods of weeks, months or years (5). The inflammatory nature of YAOS is supported by studies showing spontaneous IL-6 production by monocytes derived from the patient peripheral blood, as well as aberrant *NOD2* transcriptional levels and its signal NF-κB pathway; nevertheless, plasma levels of pro-inflammatory cytokines such as TNFa, IL-1β, IFNγ, IL-6 and IL-17 are not elevated (43). Others support the role of Th17 cells as well as latency-associated peptide-positive T cells, and not Th1/Th2 cells, in disease pathogenesis (44). Interestingly, infections and/or interferon release have been described as possible triggers, and vasoactive intestinal polypeptide as a possible *NOD2* pathway activator (45, 46).

To date, fewer than 100 patients have been described worldwide (**Supplementary Table 3**), suggesting that YAOS is underdiagnosed. Patients present with fever (60-80%), non-destructive polyarthritis/polyarthralgia (≥80%), skin disease (90%), gastrointestinal symptoms (65-90%) with no convincing endoscopic or histologic evidence of inflammatory bowel disease, serositis (10-40%), lower unilateral/bilateral extremity swelling (30-60%), sicca-like symptoms (50-60%), oral ulcers (25-50%) and eyelid swelling (up to 50%) (47–49). Rare manifestations such as ocular myositis have been also described (50, 51). Interestingly, phenotypical differences are observed among different ethnic groups, such as the lack of skin disease in patients of Asian descent (40, 52).

In our Greek cohort of 12 patients, we observed an almost equal gender distribution. In accordance to literature, all but one patient, were diagnosed in adulthood (mean age 36 years), and disease episodes lasted (mean) 8 days. We observed a higher frequency of fever, usually high grade, present in all our patients, and of serositis (58%), whereas a lower frequency of periorbital edema (25%), maculopapular rash (17%), sicca-like symptoms (8%), and lower limb swelling (8%). Interestingly, a recent study comparing 35 patients with YAOS to 28 patients with *NOD2* variants but other diagnoses, demonstrated more frequent lower leg edema and less headache in the former group (20).

With regard to YAOS treatment, glucocorticoids and sulfasalazine seem to be effective treatment options (33), however, up to half of the patients suffer major relapses despite therapy (33) (**Supplementary Table 4**). In these cases, anti-TNFa therapy seems to provide only modest or no response, while promising efficacy has been observed with the IL-1 inhibitor canakinumab (53, 54). Indeed, in a retrospective study of 7 refractory patients, all showed a complete clinical response with canakinumab starting from day 7 post treatment and reaching peak response on day 14 (53). Other drugs, such as IL-6 inhibitors, may be an option in refractory patients, nevertheless, more data are awaited to confirm their efficacy (43). In our study, despite previous favorable experience (33, 55), sulfasalazine did not completely control symptoms in both YAOS patients treated with the drug. Nevertheless, the small number of patients makes it impossible to reach a firm conclusion on the effectiveness of sulfasalazine in our patient cohort. On the other hand, in consistency with literature, more than half of our patients receiving glucocorticoids and/or IL-1 inhibitors (anakinra or canakinumab) showed a good response to treatment, in contrast to NSAIDs, colchicine and anti-TNFa agents.

Among patients with SAIDs and diagnoses other than YAOS (**Supplementary Table 5**) the frequency of *NOD2* variants varies between 3-20% (13, 16, 21, 23, 24) – 7% in our study. In particular, Demir et al. (14) observed a frequency of 6% among patients with positive genetic testing, whereas Karacan et al. (26) noted 7% among patients with at least one pathogenic variant. In accordance with these studies, we also found a frequency of 7% among patients with positive genetic testing, while 10% among patients with variants possibly influencing/causing disease. With regard to disease diagnosis, *NOD2* variants have been described among patients with mAIDs, e.g. FMF (4-10%) (10,16,18,20,24,26,27, present study), CAPS (21, 23), HIDS (21), patients with *TNFRSF1A* variants (15%) (12), *NLRP12*-related disease (13%) (21, 22, 24), VEXAS syndrome (19), as well as among patients with polygenic SAIDs such as Still’s disease (11), PFAPA (22%) (present study), Schnitzler syndrome (17), and also unclassified SAIDs (4-11% - 12% of patients with positive genetic testing) (13–15,21,23,25,26, present study), Crohn-like disease (present study) and relapsing urticaria (23, present study). In our study, FMF was the most frequent disease among patients with concurrent *NOD2* variants and a diagnosis other than YAOS; in particular, 22% of atypical FMF cases and 10% of the whole FMF cohort carried *NOD2* variants.

Interestingly, *NOD2* variants alter the phenotype of patients with the above monogenic or polygenic diagnoses, which implies a strong influence of *NOD2* in phenotypic expression beside other genes. For example, more severe disease has been observed in FMF patients carrying *NOD2* variants (10); at the same time, atypical disease features and manifestations possibly attributed to *NOD2* variants include resistance to colchicine (10,15,18,21, present study), pericarditis (21), abdominal pain (14, 16, 22) even between fever episodes (21), oral ulcers (15,22, present study) diarrhea (14, present study), Crohn’s like disease (22, present study) resistant to anti-TNFa but responsive to anti-IL-1 treatment (present study), conjunctivitis (14), erythematous rash (15, present study), and periorbital edema (present study). Interestingly, the patient with Crohn-like syndrome (**Supplementary Table 2**, patient no. 23) carried the *NOD2* G908R variant. G908R has been characterized a common coding risk variant for Crohn’s disease, nevertheless, it has been also reported in YAOS (9, 56, 57, present study). Finally, it is worth mentioning that some reports argue for the presence of intermediate entities between inflammatory bowel disease or Blau syndrome and NAIDs in patients carrying *NOD2* variants other than those usually observed in the former diseases (58–60).

With regard to *NOD2* genotype, most patients in literature, in accord with our results, carry a single gene variant in heterozygous state, that probably acts as a low penetrance variant in a gain-of-function fashion (9). In YAOS, the vast majority of cases (85%), derived from one center in the United States, carry the variant IVS8 + 158 residing in intron 8 splicing region of *NOD2* in heterozygosity; a quarter of patients also carry the variant R702W or 1007fs, residing in exon 4 and region encoding the leucin-rich repeat (31, 33, 41–43, 56). Nevertheless, several other rare variants have been previously reported. In the present study, even though our screening method did not allow examining intronic regions, we were able to detect variants already described by others, in addition to new ones (**Figure 2A**). The same was the case for patients with diagnoses other than YAOS carrying already known, but also newly detected variants (**Figure 2B**). Interestingly, patients with YAOS and those with other diagnoses shared common variants. This observation points towards the hypothesis that carriage of specific *NOD2* variants may confer susceptibility to disease by modifying or amplifying inflammation rather than triggers a specific disease (17). Indeed, the meaning of gene dosage or synergistic gene effect is of growing interest (20, 34, 44). In this context, phenotypes are mainly determined by the high penetrance variant, whereas a synergistic effect between high-low penetrance variants may create phenotypic and therapeutic variability (4).

In the current study, 18 out of 23 patients with *NOD2* variants had at least one variant located in 13 genes other than *NOD2* (67% of patients with YAOS vs. 91% other diagnoses). In a recent study (20), 44 patients were found to have coexisting -mostly digenic- variants in *NOD2* and another gene vs. 19 patients with variants in *NOD2* only (47% of patients with YAOS vs. 100% other diagnoses). Except for being more frequently diagnosed with YAOS, patients with *NOD2* variants solely, experienced more frequently chest pain. On the other hand, in agreement with the synergistic gene effect hypothesis, those carrying variants in different genes presented more often with other diagnoses or mixed clinical features. Indeed, in the present study, YAOS patients with *TNFRSF11A* and/or *MEFV* variants displayed features also compatible with TRAPS11 syndrome (36–38) and/or FMF.

In line with the literature, in the present study, the most frequently co-existing mutated gene was *MEFV*, both among patients with YAOS and those with other diagnoses. Indeed, variants in *MEFV* and *NOD2* have been also previously speculated to be synergistic (2, 18). Based on the evidence that *NOD2* contributes to disease expression in conjunction with other gene variants, Nomani et al. (20) suggested a new term, mixed NLR-associated Autoinflammatory Disease (NLR-AID), to describe mixed presentations heavily influenced by *NOD2* variants. In our cohort, we encountered a patient with SURF (patient no. 20 in **Supplementary Table 2**) with symptoms possibly attributed to *NOD2* variants, carrying a single low penetrance variant in *MEFV*, and representing, per se, a case of mixed NLR-AID. This new nosological entity remains a challenge to be further addressed in future studies.

Limitations of the present study include the following: Our screening method did not allow examining intronic gene regions that are of special interest to YAOS. Whole *NOD2* gene sequencing should be included in future studies. Nevertheless, we provided evidence on the presence of not previously described rare *NOD2* variants. Our cohort consists of a small number of patients, however, in contrast to studies with common diseases, SAIDs are exceedingly rare. Besides, due to the retrospective design of the study we might have missed information regarding clinical or therapeutic features. On the other hand, the mono-centric approach offers the benefit of enhanced standardization of the study population. Finally, one should always consider discrepancies between studies attributed to the different clinical SAID phenotypes tested, the screening methods used, number of genes tested, overlapping features between SAIDs, as well as intrinsic divergence among different populations.

To conclude, this is the first study focusing on SAIDs with *NOD2* variants, including YAOS, in a Greek patient cohort. It emphasizes the high frequency of *NOD2* variants and their impact on disease phenotype as well as the potential additive role of other coexisting gene variants. Additional studies, using whole exome or genome sequencing, to reveal the exact role of underlying gene variants in disease expression, gene-to-gene interactions that result in overlap syndromes, the type of inheritance explaining variability in penetrance and expressivity of disease, and, finally, to better describe the expanding phenotypic spectrum of *NOD2*-associated disease, are warranted.

## Data availability statement

The datasets presented in this study can be found in online repositories. The names of the repository/repositories and accession number(s) can be found in the article/**Supplementary Material**.

## Ethics statement

The studies involving humans were approved by Ethics Committee of the National and Kapodistrian University of Athens. The studies were conducted in accordance with the local legislation and institutional requirements. Written informed consent for participation in this study was provided by the participants’ legal guardians/next of kin.

## Author contributions

AK: Data curation, Writing – original draft, Writing – review & editing. OV: Data curation, Writing – review & editing. ES: Data curation, Writing – review & editing. AV: Data curation, Writing – review & editing. MT: Writing – review & editing. AG: Writing – review & editing. PS: Writing – review & editing. KL: Conceptualization, Data curation, Methodology, Project administration, Supervision, Writing – original draft, Writing – review & editing.
