# Supplementary material for: The expanding clinical spectrum of autoinflammatory diseases with NOD2 variants: a case series and literature review
Source: Front Immunol. 2024 Jan 29;15:1342668. doi: 10.3389/fimmu.2024.1342668 (PMC10859468; doi:10.3389/fimmu.2024.1342668)
Supplement: Supplementary file 1 [file Table_1.docx]

**Supplementary Table 1.** List of 26 systemic autoinflammatory disease-related genes screened by next generation sequencing and their clinical associations.

| ***Genes*** | ***Inheritance*** | ***Autoinflammatory disease*** |
| --- | --- | --- |
| *AP1S3* | AD | Pustular psoriasis |
| *CARD14* | AD | Pityriasis rubra pilaris (PRP) |
| *CECR1* | AR | Deficiency of adenosine deaminase 2 (DADA2) |
| *IL10* | AR | Early-onset inflammatory bowel disease (EO-IBD) |
| *IL10RA* | AR |  |
| *IL10RB* | AR |  |
| *IL1RN* | AR | Deficiency of IL-1 receptor antagonist (DIRA) |
| *IL36RN* | AR | Deficiency of interleukin 36 receptor antagonist (DITRA) |
| *LPIN2* | AR | Majeed Syndrome |
| *MEFV* | AR | Familial Mediterranean Fever (FMF) |
| *MVK* | AR | Mevalonate kinase (MVK) deficiency / Hyperglobulinemia D Syndrome (MKD) |
| *NLRC4* | AD | Autoinflammation with infantile enterocolitis (AIFEC) syndrome |
| *NLRP12* | AD | NLRP12-associated disorder (NLRP12-AD) |
| *NLRP3* | AD | Cryopyrin associated periodic syndromes (CAPS) |
| *NLRP7* | AR | Hydatidiform molar pregnancy (HYDM1) |
| *NOD2* | Mu/ AD | Crohn's disease (CD) / Blau syndrome (BS) - Early-onset sarcoidosis (EOS) |
| *PLCG2* | AD | APLAID/PLAID |
| *PSMB8* | AR | Nakajo-Nishimura syndrome (NNS) / Joint contractures, muscle atrophy, microcytic anemia and panniculitis-induced lipodystrophy (JMP) syndrome / Chronic atypical neutrophilic dermatosis with lipodystrophy and elevated temperature syndrome (CANDLE) |
| *PSTPIP1* | AD | Pyogenic sterile arthritis, pyoderma gangrenosum and acne (PAPA) syndrome |
| *RBCK1* | AR | Polyglucosan body myopathy, early onset, with or without immunodeficiency (PBMEI) |
| *SH3BP2* | AD | Cherubism |
| *SLC29A3* | AR | H syndrome |
| *TMEM173* | AD | STING-associated vasculopathy with onset in infancy (SAVI) |
| *TNFRSF11A* | AD | TRAPS11-syndrome |
| *TNFRSF1A* | AD | TNF receptor associated periodic syndrome (TRAPS) |
| *ELANE* | AD | Cyclic Neutropenia |

AD: autosomal dominant; AR: autosomal recessive; Mu: multifactorial

**Supplementary Table 2.** Demographics, genetic findings, diagnosis and treatment response in all 23 consecutive patients with at least one *NOD2* gene variant in the present study.

| Pt | Sex | Age at disease onset | *NOD2* | *Other Genes* | Disease diagnosis | Response to treatment | | | | | | Characteristics attributed to *NOD2* variant(s) |
| --- | --- | --- | --- | --- | --- | --- | --- | --- | --- | --- | --- | --- |
|  |  |  | Genotype and variant pathogenicity | |  | NSAIDs | GC | Colch | SFZ | TNFi | IL1i |  |
| *Patients with YAOS* | | | | | | | | | | | | |
| 1 | M | 44 | G908R/ VUS | *MEFV* V726A/ Pathogenic  *MEFV* R408Q/P369S VUS  *NLRC4* A160T/ Benign | YAOS | - | GR | GR | - | GR | GR | YAOS |
| 2 | F | 29 | R311W/ Pathogenic  R703C/ VUS | *MEFV* V726A/ Pathogenic | YAOS | - | - | - | - | - | - | YAOS |
| 3 | M | 27 | N825S/ Benign | - | YAOS | NR | - | NR | PR | PR | - | YAOS |
| 4 | M | 25 | c.*89C>T/ VUS | - | YAOS | PR | PR | PR | - | - | GR | YAOS |
| 5 | M | 42 | N289S/ Benign | *CECR1* M309I/ Benign | YAOS | PR | GR | - | - | - | GR | YAOS |
| 6 | F | 50 | A725G/ Benign | - | YAOS | PR | GR | GR | - | - | - | YAOS |
| 7 | F | 21 | R235C/ VUS | - | YAOS | GR | GR | - | - | - | - | YAOS |
| 8 | F | 30 | N409S/VUS | *IL10*  I154T/ Benign  *PLCG2* I671V/ Benign | YAOS | PR | NR | NR | - | PR | PR | YAOS |
| 9 | F | 45 | R684W/ Benign | *AP1S3* T22A/ Benign | YAOS | PR | NR | NR | - | NR | PR | YAOS |
| 10 | M | 51 | R642K/ Benign | *MEFV* A744S/ Pathogenic  *TNFRSF11A* A507T/ Benign | YAOS | - | GR | - | - | - | - | YAOS |
| 11 | M | 37 | M950L/ VUS | *PSMB8* R91G/ VUS  *TNFRSF11A* G179R/ VUS  *TNFRSF11A* D427N/ VUS | YAOS | PR | GR | GR | - | - | - | YAOS |
| 12 | M | 30 | V793M/ Benign | *CARD14 A237H*/ Benign | YAOS | GR | - | PR | - | - | - | YAOS |
| *Patients with diagnoses other than YAOS* | | | | | |  | | | | | |  |
| 13 | F | 1 | Q37R / VUS | - | PFAPA | - | GR | GR | - | - | - | Oral ulcers, diarrhea |
| 14 | F | 4 | V162I/ Benign | *MEFV* M694V/ Pathogenic  *MEFV* M694I/ Pathogenic | FMF | GR | NR | GR | - | GR | GR | Periorbital swelling, sicca-like symptoms, diarrhea |
| 15 | F | 1 | D824N/ Benign | *MEFV* L695A/ Pathogenic  *LPIN2* Q601L/ Benign  *PSTPIP1* G258A/ Benign | FMF | NA | NA | NA | NA | NA | NA | Prominent diarrhea |
| 16 | M | 1 | N289S/ Benign | *MEFV* M680I/ Pathogenic | FMF | - | - | GR | - | - | - | Prominent diarrhea |
| 17 | F | 6 | R716H/ VUS | *MEFV* M694V/ Pathogenic  *CARD14* S945T/ VUS | FMF | - | - | PR | - | - | - | Prominent diarrhea, oral ulcers, partial response to colch |
| 18 | F | 10 | A918N/ Benign | *MEFV* R761H/ Pathogenic  *TMEM173* V194L/ Benign | FMF | NA | NA | NA | NA | NA | NA | Prominent diarrhea |
| 19 | F | 4 | R471C/ Benign | *MEFV* V726A/ Pathogenic  *MEFV* M694V/ Pathogenic | FMF | - | - | GR | - | - | - | Maculopapular rash |
| 20 | F | 4 | V793M/ Benign | *MEFV* E148Q/ VUS | Undefined (SURF) | NA | NA | NA | NA | NA | NA | Oral ulcers, diarrhea |
| 21 | M | 8 | T189M/ Benign | *MEFV* R202Q/ Benign | PFAPA | GR | - | - | - | - | - | Prominent diarrhea |
| 22 | F | 23 | A292V/ VUS | *MEFV* E148Q/ VUS  *LPIN2* E601K/ Benign  *CECR1* S156L/ Benign | Urticaria with periorbital swelling | - | PR | - | - | - | - | Periorbital swelling |
| 23 | M | 31 | G908R/ VUS | *MVK* R60Pfs*31/ Pathogenic  *MVK* V377I/ Pathogenic  *CARD14* E197K/ VUS  *LPIN2* K387E/ Benign | Crohn-like syndrome | - | GR | - | - | NR | GR | No response to TNFi, complete resolution of disease activity with IL1i |

M: male; F: female; VUS: variant of uncertain significance; FMF: Familial Mediterranean Fever; PFAPA: Periodic Fever, Aphthous Stomatitis, Pharyngitis, Adenitis; YAOS: Yao syndrome; TRAPS11: *TNFRSF11A* associated hereditary fever disease; NSAIDs: nonsteroidal anti-inflammatory drugs; GC: glucocorticoids; Colch: colchicine; SFZ: sulfasalazine; TNFi: tumor necrosis factor inhibitor; IL-1i: interleukin-1 inhibitor; GR: good response; PR: partial response; NS: no response; NA: not available (lost to follow up); SURF: syndrome of undifferentiated recurrent fever

**Supplementary Table 3.** Summary of the existing evidence on Yao syndrome patient demographic and clinical characteristics. Values are expressed as means or absolute numbers.

| Reference | N | Race | Sex (M/F) | Family history | Age at Dx | Disease duration *years* | F | R | Histo / SD | A | LES | Ser | SLS | ES | OU | GI | Infl.  markers |
| --- | --- | --- | --- | --- | --- | --- | --- | --- | --- | --- | --- | --- | --- | --- | --- | --- | --- |
| Yao Q et al. 2011(3) | 7 | White | 4/3 | N | 44 | 3.1 | 4 | 6 | 6 / 2 | 5 | N | 2 | 3 | N | 1 | 3 | 5 |
| Yao Q et al. 2013 (47) | 22* | White | 9/13 | 3 | 40 | 4.7 | 13 | 19 | 8 / 3 | 20 | N | 5 | 9 | N | N | 13 | 9 |
| Yao Q et al. 2015 (41) | 54 | White | 17/37 | 4 | 45 | 10.7 | 33 | 49 | 20 / 7 | 47 | 17 | 9 | 30 | 1 | 14 | 39 | 21 |
| Yao Q et al. 2017 (33) | 52 | White | 14/38 | N | 38 | 8.8 | 35 | 47 | 52 / most | 41 | 16 | 7 | 30 | N | 14 | 34 | 14/48 |
| Navetta-Modrov B et al. 2023 (42) | 43 | Caucasian | 4/39 | NA | 41 | 12.1 | 35 | 39 | N | 35 | 27 | 18 | 27 | 21 | 21 | 39 | 27 |
| Yao Q et al. 2022 (6) | 11 | White | 1/10 | NA | 39 | 8.8 | 8 | 11 | N | 11 | 3 | 4 | 6 | 7 | N | 11 | 6 |
| Yao Q et al. 2021 (57) | 6** | 5 White, 1 Palestinian-American | 2/4 | 1 (PF) | 42 | 21 | 6 | 5 | N | 6 | N | N | 3 | N | 1 | 6 | N |
| Yao Q et al. 2013 (48) | 5 | White | 1/4 | 1 | 48 | 3.4 | 4 | 5 | 3 / 1 | 4 | 5 | 1 | 3 | N | N | 3 | NA |
| Yang X et al. 2018 (52) | 3 | Han | -/3 | N | 43 | 14.3 | 3 | N | N | 2 | 1 | N | 1 | 1 | 1 | 2 | 3 |
| Trueb B et al. 2021 (45) | 1 | NA | 1/- | N^***^ | NA | 14 | Y | Y | N | Y | N | N | Y | N | N | LF | NA |
| Yao Q et al. 2014 (44) | 1 | White | 1/- | NA | 23 | 23 | Y | Y | Y / Y | N | N | N | N | N | N | Y | Y |
| Estephan M et al. 2017 (55) | 1 | White | 1/- | NA | 23 | 0.5 | Y | N | N | Y | N | N | N | N | N | N | Y |
| Navetta-Modrov B et al. 2021 (46) | 1 | Caucasian | -/1 | Y | 39 | 1 | Y | Y | Y / N | Y | Y | N | Y | N | N | Y | N |
| Esse I et al. 2023 (51) | 1 | NA | -/1 | Y^***^ | 16 | 3 | Y | Y | Y / N | Y | N | N | N | N | N | Y | NA |
| Qin W et al. 2020 (40) | 1 | Han | -/1 | N | 42 | 3 | Y | N | N | Y | N | N | N | N | N | Y | NA |
| Hua Y et al. 2019 (24) | 3 | Han | -/3 | N | 45 | 9 | 3 | N | N | 2 | N | N | N | 1 | 1 | 3 | NA |
| Yao Q. 2020 (56) | 3 | Caucasian | -/3 | 1 | 50 | 21.6 | 3 | 3 | N | 3 | N | N | 2 | N | N | 3 | N |
| Brailsford CJ et al. 2022 (54) | 1 | White | 1/- | N | 27 | NA | Y | Y | Y / N | Y | N | N | N | N | N | Y | N |
| Yzeiraj E et al. 2016 (49) | 1 | NA | -/1 | NA | 53 | 2 | Y | Y | N | Y | N | Y | Y | N | N | Y | Y |
| Yao Q et al. 2023 (53) | 7 | White | 3/4 | NA | 40 | 20 | 7 | 6 | N | 6 | N | 6 | 5 | N | N | 7 | 5 |
| Nomani H et al. 2023 (20) | 35 | Caucasian | 2/33 | NA | 45 | 6 | 22 | 31 | N | 31 | 24 | 11 | 24 | 16 | 12 | 30 | 12 |
| McDonald C et al. 2018 (43) | 1 | White | 1/- | NA | 67 | 13 | Y | Y | N | Y | N | N | N | N | N | Y | Y |
| Present study | 12 | White, 1 Armenian included | 7/5 | N | 42 | 6 | 12 | 5 | 3/N | 8 | 1 | 7 | 1 | 3 | 3 | 7 | 11 |

M: male; F: female; Dx: diagnosis; N: no; Y: yes; NA: not available; PF: periodic fever; F: fever; R: rash; Histo: skin histological examination; SD: spongiotic dermatitis; A: arthritis/arthralgia; LES: lower extremity swelling; Ser: serositis and/or chest pain; SLS: sicca-Like symptoms; ES: eyelid swelling; OU: oral Ulcers; GI: gastrointestinal symptoms; Infl. markers: inflammatory markers; LF: liver fibrosis

* 7/22 included in Ref. 3; ** FMF + YAOS, n=1; ^***^ same *NOD2* variation in ancestors

**Supplementary Table 4.** Summary of the existing evidence on treatment response in patients with Yao syndrome.

| Reference | N | NSAIDs | GC | HCQ | MTX | Sulfasalazine | Colchicine | TNFi | IL-6i | ANA | CAN |
| --- | --- | --- | --- | --- | --- | --- | --- | --- | --- | --- | --- |
| Yao Q et al. 2011 (3) | 7 | ΝR^#^ | GR^#^ | 6 NR | 1 NR | 2 PR | 1 NR | 1 PR | - | - | - |
| Yao Q et al. 2013 (47) | 22* | NR^#^ | GR^#^ | NR^#^ | NR^#^ | GR^#^ | NR^#^ | NR^#^ | - | - | - |
| Yao Q et al. 2015 (41) | 54 | NR of joint symptoms^#^ | GR of joint symptoms^#^ | - | - | GR of joints symptoms^#^ | - | 1/1 PR | 1/1 PR | - | 1/1 GR |
| Yao Q et al. 2017 (33) | 52 | 46/46 NR | 16/19 GR  3/19 PR | NR^#^ | NR^#^ | 11/22 GR  3/22 PR  2/22 NR  6/22 side effects | - | 2/3 NR  1/3 PR | 1/1 GR | 2/2 PR  1/2 side effects | 2/2 PR |
| Yao Q et al. 2021 (57) | 6 | - | - | - | - | 1 GR  1 PR** | 1 GR  3 PR  1 NR** | - | - | - | - |
| Yao Q et al. 2013 (50) | 1 | - | GR | - | - | - | - | - | - | - | - |
| Yang X et al. 2018 (52) | 3 | - | 3 GR | - | 1 GR | 2 GR | - | - | - | - | - |
| Trueb B et al. 2021 (45) | 1 | - | GR | - | - | - | - | GR | GR | - | - |
| Yao Q et al. 2014 (44) | 1 | - | GR | - | - | - | - | - | - | - | - |
| Estephan M et al. 2017 (55) | 1 | - | - | - | - | GR | NR | - | - | - | - |
| Navetta-Modrov B et al. 2021 (46) | 1 | - | - | - | - | NR | - | - | - | - | GR |
| Esse I et al. 2023 (51) | 1 | - | NR | - | - | NR | - | - | - | - | - |
| Hua Y et al. 2019 (24) | 3 | - | 3 GR | - | 2 GR | - | - | - | - | - | - |
| Yao Q. 2020 (56) | 3 | - | - | - | - | - | 1 GR  2 PR | - | - | - | - |
| Brailsford CJ et al. 2022 (54) | 1 | - | NR | - | - | NR | NR | - | - | - | - |
| Yzeiraj E et al. 2016 (49) | 1 | NR | PR | - | - | - | NR | - | - | - | GR |
| Yao Q et al. 2023 (53) | 7 | - | NR^#^ | - | - | NR^#^ | - | - | - | - | 7 GR |
| McDonald C et al. 2018 (43) | 1^$^ | - | PR | - | NR | - | - | NR | GR | NR | - |
| Present study | 12 | 2GR  6 PR  1 NR | 6 GR  1 PR  3NR | - | - | 0/2 NR | 2GR  3 PR  3 NR | 1GR  2 PR  1 NR | - | 3 GR  1 NR^##^ | 2 PR |

NSAIDs: nonsteroidal anti-inflammatory drugs; GC: glucocorticoids; HCQ: hydroxychloroquine; MTX: methotrexate; TNFi: tumor necrosis factor inhibitor; IL-6i: interleukin-6 inhibitor; ANA: anakinra; CAN: canakinumab; NR: no response; GR: good response; PR: partial response; NA: not available

MI: minimal improvement; PR: partial response; AR: adverse reaction; TR: transient response; UsR: unsatisfactory response

^#^ absolute number of patients unknown; * 7/22 included in Ref. 3; ** Overlap FMF + YAOS; ^##^ this patient also received CAN; ^$^NR also to azathioprine, mycophenolate, leflunomide, cyclosporine, abatacept, rituximab

**Supplementary Table 5.** Summary of the existing evidence on the genotype and phenotype of patients with autoinflammatory diseases other than Yao syndrome carrying *NOD2* variants.

| Reference | No. of pts with SAIDs assessed | No. of genes screened | Pts with *NOD2* variants | Phenotype | | *NOD2* variants | Coexisting *MEFV* variants and other mutated genes |
| --- | --- | --- | --- | --- | --- | --- | --- |
| Berkun et al. 2012 (10) | 103 children with FMF | 2  Only G908R and R702W variations screened | 10 | Pts with *NOD2* variants had more erysipelas-like rash, acute scrotum attacks, colchicine resistance, severe disease | | All heterozygous  9 G908R  1 R702W | NA |
| Garcia-Melchor et al. 2014 (11) | 18 pts with adult-onset Still’s disease | 2 | exact number NA (≥4) | NA | | R702W/-  R791Q/-  R459R  R587R  P268S | *NLRP3* |
| Ueda et al. 2016 (12) | 27 pts with SAIDs and *TNFRSF1A* variants | unknown | 4 | Female, 12 yrs at onset, attacks 10-16d, fever, myalgia, rash, conjunctivitis, arthralgias, elevated CRP, GR to NSAIDs | | R471C/- | *TNFRSF1A* heterozygous  *PSMB8* heterozygous |
|  |  |  |  | Male 20 yrs at onset, attacks 30d, fever, hepatosplenomegaly, elevated CRP, response to NSAIDs/colchicine | | A612T/- | *TNFRSF1A* heterozygous  *MEFV* E148Q/-  PSMB8 heterozygous |
|  |  |  |  | Male, 47 yrs at onset, attacks 7d, fever, abdominal pain, myalgias, arthralgias, rash, elevated CRP, response to GC+, colchicine ++ | | R471C/- | *TNFRSF1A* heterozygous  PSMB8 heterozygous |
|  |  |  |  | Female, 32 yrs at onset, attacks 0.5-5d, fever, myalgias, arthralgias, chest pain, headache, elevated CRP, GR to NSAIDs, GC, colchicine | | R471C/- | *TNFRSF1A* heterozygous  *MEFV* G304R/- |
| Rusmini et al. 2016 (21) | 50 pts with SAIDs | 10 | 9  Clinical information available in 7 pts | CAPS | CAPS typical | G908R/- | *NLRP3* heterozygous |
|  |  |  |  |  | CAPS-like | R791Q/- | *NLRP3* heterozygous |
|  |  |  |  |  | Atypical CAPS, abdominal and chest pain, pericarditis, arthralgias, urticarial rash, elevated CRP, GR to IL1i | T294S/- | *NLRP3* double heterozygous  *LPIN2* heterozygous |
|  |  |  |  | Classical *NLRP12* RD | | 1007fs/SNP13 | *NLRP12* heterozygous |
|  |  |  |  | FMF resistant to colchicine, persistent abdominal pain even between fever episodes | | G908R/- | *MEFV* R761H/M680I |
|  |  |  |  | Undefined – fever, arthromyalgias in pelvic region | | N289S/- | *MVK* double heterozygous |
|  |  |  |  | Classical HIDS | | N289S/- | *MVK* homozygous |
| Kostik et al. 2018 (22) | 15 children with NLRP12 RD | 302 | 2 | Periodic fever, aphtous stomatitis, abdominal pain | | p.L1007fs/- | *NLRP12* heterozygous  *NLRP3* heterozygous |
|  |  |  |  | Periodic fever, abdominal pain, splenomegaly, Crohn’s-like disease | | p.L1007fs/- | *NLRP12* heterozygous |
| Hoang et al. 2018 (23) | 27 pts with SAIDs | unknown | 3 | Undefined – female, fever every 2-4w, irritability, insomnia, nausea/vomiting, arthralgias, GR to steroids | | c.2017 C>T/- | - |
|  |  |  |  | Muckle-Wells – male, fever, urticaria, sinus congestion, deafness, multiple sclerosis features | | c.2407 C>A/- | - |
|  |  |  |  | Male with Crohn’s and cold-induced urticaria, treatment with TNFi and sulfasalazine | | c.3017 dupC/- | - |
| Hua et al. 2019 (24) | Total 68  41 (+) *vs.* 27 (-) genetic result  50 with definite SAID diagnosis after genetic testing | Whole exome | 10 (3 pts with YAOS and 3 Blau syndrome) | 2/13 FMF  2/10 NLRP12 RD | | A699T/- | *MEFV* G304R/- |
|  |  |  |  |  |  | R471C/- | *MEFV* G304R/- |
|  |  |  |  |  |  | R471C/- | *NLRP12* double heterozygous |
|  |  |  |  |  |  | R471C/- | *NLRP12* double heterozygous |
| Ter Haar et al. 2019 (25) | 9 pts with undefined SAID | unknown | 1 | Undefined disease | | R702W/SNP8 | NA |
| Karacan et al. 2019 (26) | 41 pts with SAIDs and at least one pathogenic variant | 15 | 3 | FMF, female 3 yrs old | | E970G/- | *MEFV* M694V/- |
|  |  |  |  | Undefined, male 9 yrs old | | F677L/- | *LPIN2* homogygous |
|  |  |  |  | FMF, male 11 yrs old | | R426H/- | *MEFV* R761H/-  *PLCG2* heterozygous |
| Fujimoto et al. 2020 (27) | 22 pts with FMF | 11 | 2 | Male, 39 y.o., with fever, abdominal pain, arthralgia, NO diarrhea, thoracic pain, myalgias, ulcers, rash | | frameshift | *MEFV* 694I/E148Q |
|  |  |  |  | Female, 33 y.o., fever, arthralgia | | R471C | *MEFV* E148Q/L110P |
| Bozgeyik et al. 2020 (28) | 182 pts with FMF  28 pts with *MEFV*+other gene variation | 17 | 8 | FMF | | T91A/-, L1007fs/-, G908R/- (n=3), R439C/-, N852S/-, R702T/G908R | 8/8 *MEFV*  M680I homozygous; M694V/V726A (n=2); M694V/-; M694V/E148Q; E148Q/- (n=2); S104C/-  1/8 *CARD14* heterozygous  1/8 *TNFRSF11A* heterozygous |
| Sözeri et al. 2020 (13) | 71 pts with SAID phenotype  Half of them with undefined disease after genetic testing  15 with (-) genetic testing | 16 | 2 | Undefined disease | | E441K/- | - |
|  |  |  |  |  |  | L682F/- | *MEFV* A761H/- |
| Demir et al. 2020 (14) | 64 clinically unclassified SAIDs  49 unclassified after genetic testing  36 (+) *vs.* 28 (-) genetic result | 16 | 2 | Undefined (male, onset 4 yrs, 3-day attacks, 12 attacks/yr, fever, conjunctivitis, abdominal pain, diarrhea, arthralgia) | | R541W/- | - |
|  |  |  |  | CAPS (male with recurrent fever, urticarial rash and arthralgia, onset 1 month) | | R426H/- | *NLRP3* heterozygous |
| Papa et al. 2020 (15) | 50 pts with clinically undefined SAIDs  9 pts no variants  45 pts with undefined disease after genetic testing | 41 | 5 | Nodular panniculitis, fever, arthralgia, PR to anakinra, GR to TNFi and GC | | R684W/- | *CARD14* double heterozygous  *NLRC4* heterozygous  *NLRP3* heterozygous  *NLRP7* heterozygous |
|  |  |  |  | Fever, abdominal pain, headache, arthralgia, PR to colchicine | | G908R/- | *ADAR1* heterozygous  *CARD14* heterozygous |
|  |  |  |  | Fever, malaise, exudative pharyngitis, erythematous rash, arthromyagia, lymphadenopathy, hepatosplenomegaly, elevated ferritin and transaminases, GR to GC | | P427L/- | - |
|  |  |  |  | Fever, polyarthritis, erythematous rash, GR to GC | | I836T/- | *MEFV* V726A/- |
|  |  |  |  | Fever 3d every 3 weeks, aphtosis, maculopapular rash, arthralgia, PR to colchicine | | A725G/- | *CARD14* heterozygous |
| Hidaka et al. 2020 (16) | 179 pts with unexplained fever  Among them 43 pts with FMF | 11 | 7 | 2 FMF | | A110T/- | *NLRP12* heterozygous |
|  |  |  |  |  |  | unknown | *MEFV* M694I/?  Other genes unknown |
|  |  |  |  | 5 non-FMF pts | | unknown | unknown |
|  |  |  |  | Fever 7/7, Abdominal pain 2/7, thoracic pain 2/7, arthralgia 2/7, myalgia 3/7, erysipelas-like rash 2/7 | | 5 missense  1 frame shift  1 nonsense | *MEFV*, *NLRP12*, other unknown |
| Navetta-Modrov and Yao 2021 (17) | Schnitzler syndrome | 6 | 1 | Female 51 y.o. with bilateral leg swelling, diarrhea, arthralgias, urticaria, IgMλ monoclonal gammopathy, elevated ESR | | IVS8+158/1007fs | *TNFRSF1A* double heterozygous |
| Lee et al. 2022 (18) | Case report | 10 | 1 | FMF-like, woman, onset 43 yrs, fever 1-9d every 4-5d, abdominal pain, lymphadenopathy, acute phase response, resistance to colchicine, GR to tocilizumab | | V92P/- | *MEFV* L508V/- |
| Guzman Rivera et al. 2022 (19) | VEXAS syndrome | 2 | 1 | Male 75 y.o. with fever, sore throat, abdominal pain and diarrhea, negative colonoscopy, periorbital swelling, arthritis, maculopapular rash, acute phase response, GR to GC and anakinra | | IVS8+158/R702W | *UBA1* |
| Nomani et al. 2023 (20) | 63 pts with *NOD2* variants | 6 | 63 (36 pts with YAOS) | 2 FMF | | A725G/- | *MEFV* E148Q/- |
|  |  |  |  |  |  | IVS8+158/- | *MEFV* E148Q/P369S/R408Q |
|  |  |  |  | 25 mixed NAID | | IVS8+158/-; 1007fs/-; IVS8+158/1007fs; IVS8+158 homozygous/1007fs; P668L/-; IVS8+158/R702W; R87C/-; V955I/-; IVS8+158/V955I; S431L/V793M; G908R/-; R791Q/- | *MEFV* M694V/-; P369S/R408Q; K695R/-; R329H/-; A744S/-; V726A/-; I591T/-; E148Q/P369S/R408Q  *NLRP12*  *NLRP3*  *TNFRSF1A* |

Pt: patient; SAID: systemic autoinflammatory disease; *NLRP12* RD: *NLRP12* related disease; FMF: Familial Mediterranean Fever; CAPS: cryopyrin-associated periodic syndrome; HIDS: Hyperimmunoglobulinemia D with periodic fever syndrome; NAID: *NOD2*-associated autoinflammatory disorder; YAOS: Yao syndrome; NSAIDs: nonsteroidal anti-inflammatory drugs; GC: glucocorticoids; TNFi: tumor necrosis factor inhibitor; IL1i: interleukin-1 inhibitor; CRP: C-reactive protein; ESR: erythrocyte sedimentation rate; GR: good response; PR: partial response; NA: not available
